# Stereotactic body radiotherapy plus transcatheter arterial chemoembolization for inoperable hepatocellular carcinoma patients with portal vein tumour thrombus: A meta-analysis

**DOI:** 10.1371/journal.pone.0268779

**Published:** 2022-05-20

**Authors:** Xiao-fei Zhang, Lin Lai, Hui Zhou, Yuan-jun Mo, Xu-quan Lu, Min Liu, Yun-xin Lu, En-cun Hou

**Affiliations:** 1 Department of Oncology, Ruikang Hospital Affiliated to Guangxi University of Chinese Medicine, Nanning, China; 2 Department of Radiotherapy, Tumor Hospital of Guangxi Medical University, Nanning, China; Cincinnati Children’s Hospital Medical Center, UNITED STATES

## Abstract

**Background:**

The efficacy and safety of stereotactic body radiotherapy (SBRT) plus transcatheter arterial chemoembolization (TACE) versus SBRT or TACE alone(monotherapy) for hepatocellular carcinoma (HCC) patients with portal vein tumour thrombus (PVTT) remains controversial. This meta-analysis was performed to provide more powerful evidence for clinical strategies in inoperable HCC with PVTT.

**Methods:**

We searched the PubMed, EMBASE, Web of Science, Cochrane Library, China Biology Medicine (CBM), China National Knowledge Infrastructure (CNKI), VIP Journal Integration Platform (VIP), and WanFang databases for eligible studies. We pooled the results of 1- and 2-year overall survival rates (OSRs), objective response rates (ORRs), and adverse events (AEs) between the two groups and performed a subgroup meta-analysis for study type, control group, treatment order, and the interval between SBRT and TACE.

**Results:**

Nine studies with 10 cohorts involving 938 patients were included in our meta-analysis. SBRT plus TACE yielded significantly higher 1-year OSR (RR, 1.52[95% CI, 1.33–1.74]), 2-year OSR (RR, 2.00 [95% CI: 1.48–2.70]), ORR (RR = 1.22 [95% CI, 1.08–1.37]), and a lower progression disease (PD) rate (RR = 0.45 [95% CI:0.26–0.79]) than monotherapy. No significant differences were detected in CR, PR, SD, or AEs between the two groups. Subgroup analysis regarding study type, control group, and treatment order indicated that compared with monotherapy, the combination of SBRT with TACE was associated with an increase in 1- and 2-year OSRs but not in ORR. In regard to the interval between SBRT and TACE, subgroup analysis found that the combination therapy for patients with an SBRT-TACE interval <28 days was preferable to monotherapy in the 1- and 2-year OSRs, and ORR. However, for patients with an SBRT-TACE interval ≥28 days, no obvious distinctions were observed in the 1-year OSR, 2-year OSR, or ORR between the two groups.

**Conclusion:**

The combination of SBRT with TACE appears to be better than monotherapy in treating HCC with PVTT and should be recommended for inoperable HCC patients with PVTT.

## Introduction

Worldwide, hepatocellular carcinoma (HCC) was the seventh most commonly diagnosed malignancy and the third leading cause of cancer-related death in 2020 [1]. Portal vein tumour thrombus (PVTT), regarded as the most usual form of vascular invasion in liver cancer, is observed in 10–60% of HCC patients at the time of diagnosis [2,3]. Given that PVTT is associated with portal vein hypertension, varix or ascites formation, hepatic dysfunction, and dissemination of tumour cells, the prognosis of HCC with PVTT remains grave, with a median overall survival (mOS) of merely 2–4 months under supportive care [4–6]. What’s more, there are no extremely effective treatment choices for inoperable HCC with PVTT thus far.

HCC with PVTT is fallen into Barcelona Clinic Liver Cancer (BCLC) stage C, and sorafenib is usually regarded as the first-line therapy for patients with PVTT in accordance with the BCLC guidelines for liver cancer [5,7]. Some randomized controlled trials (RCTs) have demonstrated that nearly 3-month survival time of HCC patients with PVTT can be prolonged by sorafenib [5,8,9]. However, unsatisfactory clinical efficacy and potential complications warrant exploration of other treatment modalities. Liver surgery is only suitable for patients with excellent hepatic function, a completely resectable primary tumour, and no extrahepatic metastases [10–12]. Transcatheter arterial chemoembolization (TACE) was firstly regarded a contraindication for HCC with PVTT located in the trunk or first branch of the portal vein, due to the possibility of hepatic ischaemic necrosis from vascular obstruction [6,13]. Subsequently, many studies have shown that TACE could be safe and more effective than palliative care for some highly selected HCC with PVTT. [11,14,15].

Radiotherapy (RT) was also demonstrated to play a huge role in killing malignant cells and the recanalization of PVTT occlusion [16,17]. Nevertheless, conventional fractionated radiotherapy (CFRT) for HCC is restricted owing to the low tolerance of liver tissue for radiotherapy and the potential risk of radiation-induced liver disease (RILD) [18]. With the development of radiotherapy techniques and the progressive understanding of the maximum liver tolerated dose, stereotactic body radiation therapy (SBRT) has been increasingly applied for HCC with PVTT with the advantage of concentrating high-dose radioactive rays precisely on the target lesion, thus sparing the normal liver tissue at risk from high doses of radiation and reducing the incidence of hepatotoxicity to some extent [19,20]. Many studies have shown the preferable survival benefits of SBRT for HCC with PVTT [21–23]. A retrospective study [22] reported that local progression-free survival (LPFS) rate and 1-year OSR of patients with PVTT in the SBRT group were 69.6% and 34.9%, respectively, significantly better than those in the CFRT group (32.2% and 15.3%). Moreover, the incidence of RILD in SBRT group was marginally lower than that in CFRT group (16.7% vs. 19.8%, *p* = 0.646). Matsuo et al. concluded that the 1-year OSR of 49.3% in SBRT for HCC patients with PVTT was significantly higher than that in 3-DCRT (29.3%) [23]. However, the effectiveness of SBRT monotherapy is still insufficient, and a combination of other treatment modalities, such as TACE, HAIC, microwave ablation or targeted drugs, is required to further improve the ORR and OSR.

It was reported that the combination of SBRT and TACE might be an excellent choice for HCC with PVTT than SBRT or TACE alone (monotherapy) [24,25]. Choi et al. retrospectively analyzed the outcomes of SBRT combined with or without TACE in patients with HCC and PVTT, the results revealed that patients treated by SBRT plus TACE had better ORR and 1-year survival rate than those treated by SBRT alone (56.3% VS. 50% and 71.4% VS. 14.6%, respectively) [24]. The similar results were also reported by Kang et al [25]. They found that 1- and 2-year survival rates were higher in patients with SBRT plus TACE (58.8% and 29.4%) than in patients with SBRT alone (50.0% and 23.3%). However, no large randomized controlled trial has reported the efficacy of SBRT combined with TACE in the treatment of HCC with PVTT. Herein, we performed this meta-analysis for the evaluation of the effectiveness and security of SBRT plus TACE versus monotherapy in inoperable HCC with PVTT.

## Materials and methods

### Literature search

The current meta-analysis was conducted in accordance with the Preferred Reporting Items for Systematic Reviews and Meta Analyses (PRISMA) Statement [26]. An integrated literature search was performed through the PubMed, Cochrane Library, EMBASE, Web of Science, China Biology Medicine (CBM), China National Knowledge Infrastructure (CNKI), VIP Journal Integration Platform (VIP), and WanFang databases from the inception dates of the databases to July 1, 2021. The search terms were as follows: (“hepatocellular carcinoma” or “hepatoma” or “liver cancer” or “liver neoplasm” or “HCC”) AND (“portal vein tumour thrombus” or “portal vein thrombosis” or “PVTT”) AND (“stereotactic body radiotherapy” or “stereotactic radiotherapy” or “stereotactic radiosurgery” or “SBRT” or “cyberknife” or “gamma knife”) AND (“transcatheter arterial chemoembolization” or “transarterial chemoembolization” or “TACE”). Besides, all references of the included articles were also manually searched to identify other potentially eligible studies.

### Selection criteria

The included studies complied with the following criteria: (1) the subjects were inoperable HCC patients with PVTT without metastases, confirmed pathologically or diagnosed by magnetic resonance imaging (MRI) or computed tomography (CT); (2) the included studies consisted of a treatment group treated with SBRT combined with TACE and a control group treated with SBRT or TACE alone (monotherapy); (3) detailed data on 1-year survival rate, 2-year survival rate, complete response (CR), partial response (PR), stable disease (SD), progressive disease (PD), objective response rate (ORR), and adverse effects (AEs); (4) study type described as randomized controlled trials (RCTs) or non-randomized controlled trials (NRCTs); (5) the language was restricted to English or Chinese. The literature meeting any of the criterion below were excluded: (1) literature reviews, meta-analysis, case reports, comments, letters, conference proceedings or abstracts, animal experiments; (2) without data available, or duplicated data; or (3) the absence of a control group.

### Data extraction

Two authors extracted all eligible data in accordance with the inclusion and exclusion criteria as mentioned above independently. Any conflict during the data extraction was settled through discussion or by consultations with the corresponding author. The following information was extracted using a standardized form: (1) Basic features of the included studies, such as first author, publication year, country, study design, sample size, age, gender, performance status score, Child-Pugh class, tumour stage, and the type of PVTT. (2) Intervention characteristics: treatment modalities, radiation dose and fraction, interventional chemoembolization drugs and dose. (3) Outcomes: 1- and 2-year survival rates, ORR, and AEs. Survival rates were either described in the original literature or extracted from the survival curves using Engauge Digitizer 6.1 software [27]. Tumour target was defined as thrombus and primary tumour. The evaluation of tumour response rates was performed on the basis of the modified Response Evaluation Criteria In Solid Tumours (mRECIST) for HCC [28]. Complete response (CR): full regression of the tumour lesions; partial response (PR): more than 30% reduce in the longest diameters of the tumour lesions; progressive disease (PD): more than 20% growth in the longest diameters of target lesions; stable disease (SD): all other variations; objective response rate (ORR) = CR + PR.

### Quality assessment

Two researchers conducted a quality evaluation of the included studies independently. The Cochrane assessment tool was employed to assess the quality of every RCT for risk of bias from the following six dimensions: production of random sequences, distribution concealment, blinding, incomplete result data, selective reporting, and other biases [29]. The quality of each NRCT was evaluated by the Newcastle–Ottawa scale (NOS) [30], which involves the following three main indicators: comparability, selection, and result evaluation. The quality of the studies was classified into three levels: high (≥7 points), medium (4–6 points), and low quality (≤3 points).

### Statistical analysis

We conducted all the meta-analysis using Review Manager Statistical Software (RevMan Version 5.3, Nordic Cochrane Centre, Oxford, England) and calculated the risk ratios (RRs) with the corresponding 95% confidence intervals (CIs) in regard to 1- and 2-year survival rates, tumour target lesion response, and the occurrence rates of AEs. Heterogeneity was assessed by *I*^*2*^ statistics and the chi-square test [31]. In case of no obvious heterogeneity (*I*^*2*^ ≤ 50% and *p*>0.1), the effect sizes was merged with a fixed-effects model; however, in case of obvious heterogeneity (*I*^*2*^ >50% and *p*≤ 0.1), a random-effects model was applied. A funnel plot was employed to evaluate potential publication bias, and the symmetry of the funnel plot was quantitatively analysed by Egger’s test [32]. A *p* value < 0.05 was of statistical significance.

## Results

### Search results and basic features of the included studies

**Fig 1:**
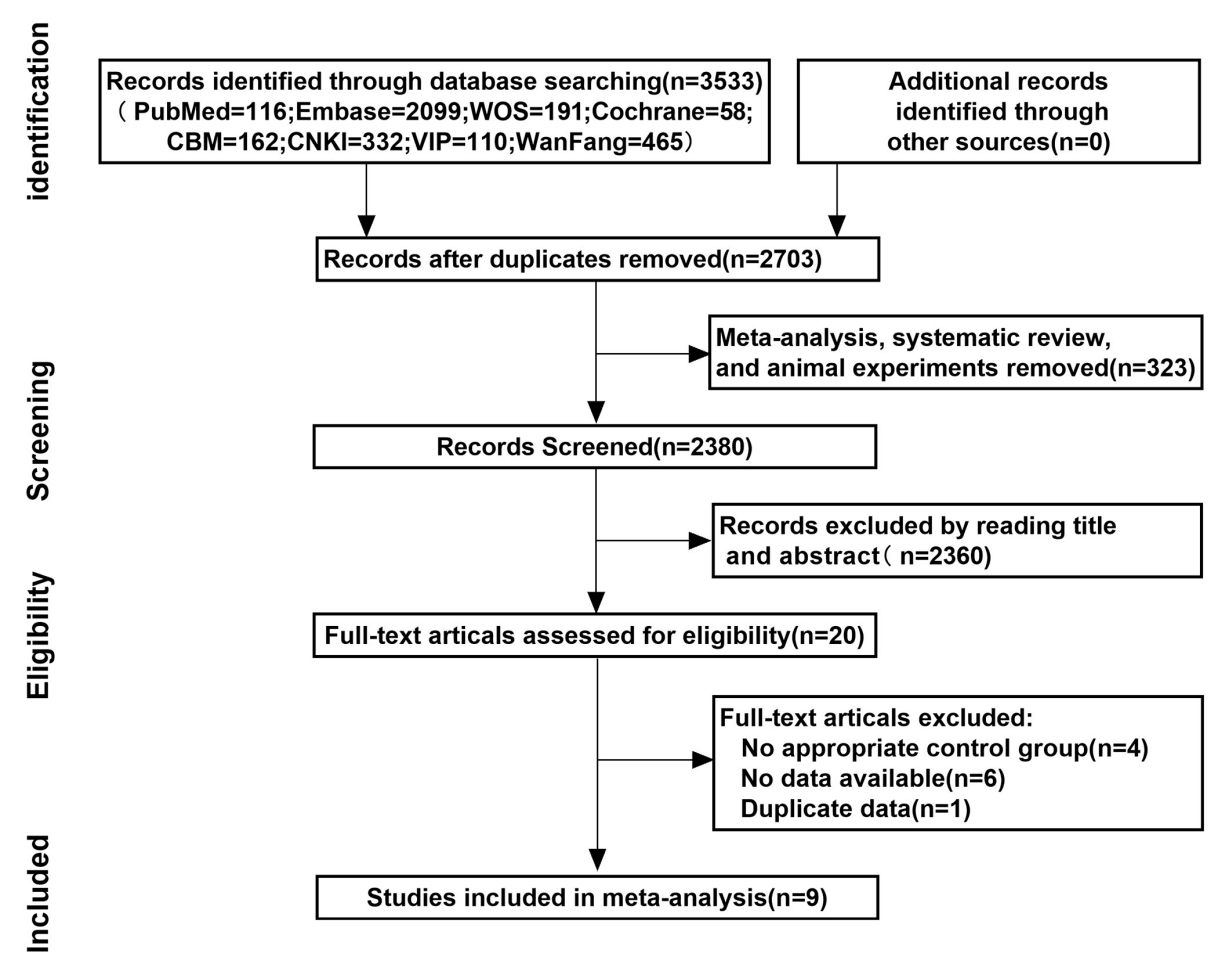
Flow chart of literature screening.

There were 3533 relevant studies initially identified through the systematic literature search. Twenty studies were selected for possible inclusion in our meta-analysis after all of the titles and abstracts were screened. Eleven articles were excluded due to duplication or not satisfying the inclusion and exclusion criterion after reading the full text carefully. Ultimately, 9 studies with 10 cohorts were included in the present review (455 patients in the SBRT plus TACE group and 483 patients in the monotherapy group). The flow chart of the literature screening selection is shown in Fig 1.

Of these 9 studies included, 4 were RCTs, and 5 were non-RCTs. Eight were conducted in China, while only one was conducted in South Korea. Kang et al.’s study [25] involved two cohorts: Kang 1 (SBRT followed by TACE vs. SBRT alone) and Kang 2 (TACE followed by SBRT vs. SBRT alone). The control group in 5 studies was SBRT alone and that in the remaining 4 studies was TACE alone. For the combined treatment group, SBRT followed by TACE was performed in 5 of 10 cohorts, while TACE followed by SBRT was performed in the other 5 cohorts (Table 1).

**Table 1:** Basic features of the included studies.

Study	Country	Study type	Groups	Gender (M/F)	Age (median, range) (mean ± SE)	ECOG PS	Stage	CPS A/B/C		PVTT type	End points
Choi,2020 [24]	Korea	NRCT	S+T vs. S	20/4	56(42–78)	0/1	III/IV		A/B	Trunk/Branch	OS, ORR
Lu,2016 [33]	China	NRCT	T+S vs. T	376/65	NA	0/1/2	NA		A	Trunk/Branch	OS
Shui,2018 [34]	China	NRCT	S+T vs. S	59/11	53.8(25–75)	0/1	NA		A/B/C	Trunk/Branch	OS
Kang1,2014 [25]	China	NRCT	S+T vs. S	44/20	53(19–79)	NA	Ⅱb/Ⅲa		A/B	Trunk/Branch	OS, ORR
Kang2,2014 [25]	China	NRCT	T+S vs. S	46/21	53(19–79)	NA	Ⅱb/Ⅲa		A/B	Trunk/Branch	OS, ORR
Zhu,2014 [35]	China	RCT	T+S vs. T	54/30	44.6 ± 3.5	NA	NA		A/B	NA	OS
Han,2015 [36]	China	RCT	S+T vs. S	34/36	48	NA	Ⅱb/Ⅲa		NA	NA	OS, ORR
Zhan,2012 [37]	China	RCT	S+T vs. S	51/45	42.6(24–73)	NA	NA		NA	NA	OS, ORR
Zhou,2019 [38]	China	NRCT	T+S vs. T	42/22	50	0/1/2	Ⅱa/Ⅱb		A/B	Trunk/Branch	OS, ORR
Zhang,2020 [39]	China	RCT	T+S vs. T	47/33	52	NA	NA		A/B	NA	ORR

S+T:SBRT followed by TACE;T+S:TACE followed by SBRT;S:SBRT;T:TACE; PVTT: Portal vein tumour thrombosis; OS: Overall survival; ORR: Objective response rate; RCT: Randomized controlled trial; NRCT: Non-randomized controlled trial; CPS: Child-Pugh Score; NA: Not available.

### Quality evaluation of the included studies

**Fig 2:**
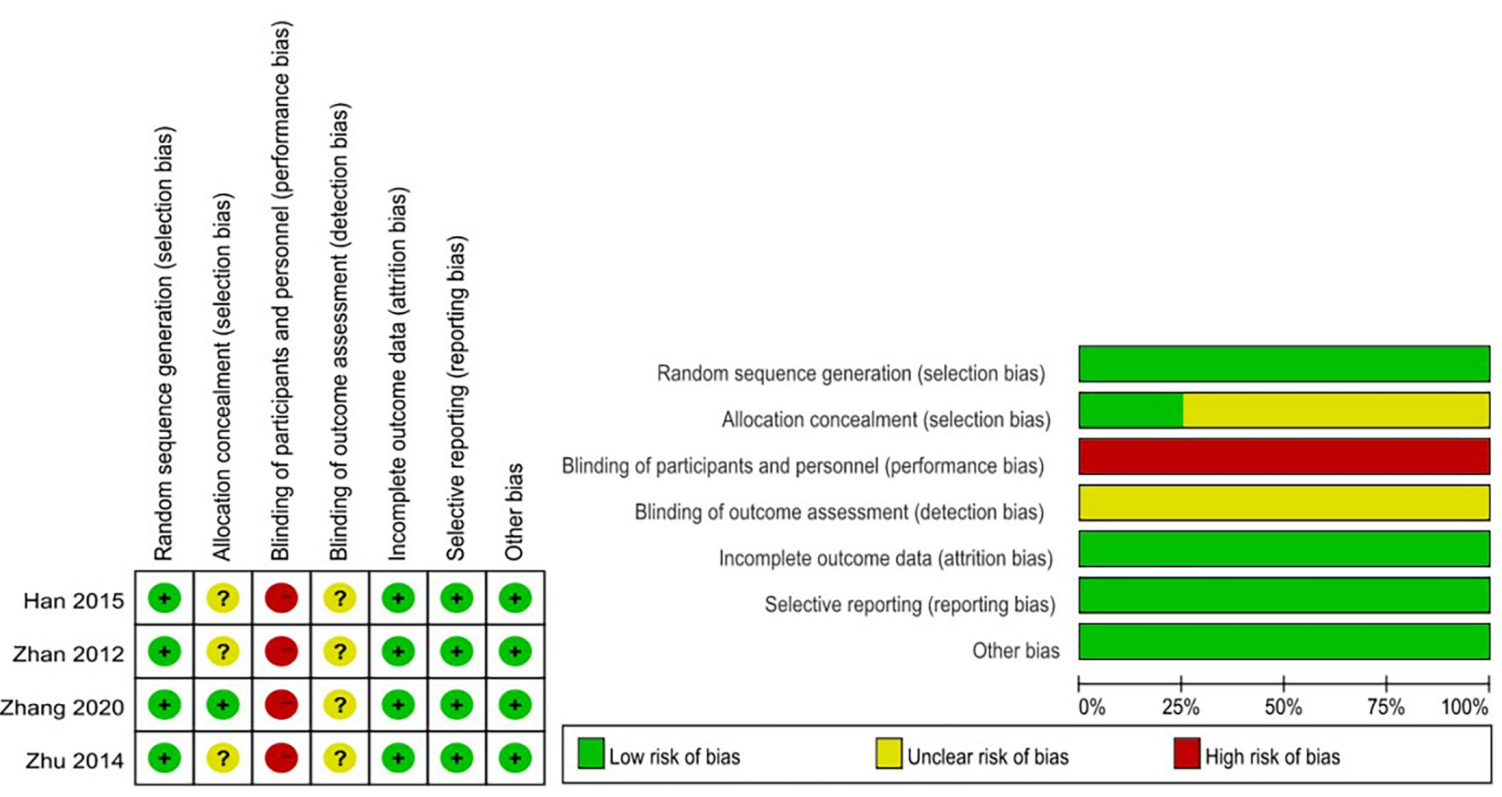
Risk of bias of RCTs studies.

In terms of quality, among all 5 non-RCTs, 2 scored 7 points and 3 scored 8 points (Table 2). Four RCTs were at moderate risk of bias (Fig 2). Overall, 9 included studies were of medium to high quality.

**Table 2:** Quality evaluation of non-RCTs studies by Newcastle–Ottawa scale.

Study	Selection				Comparability	Outcome			NOS Score
	a	b	c	d	e	f	g	h	
Choi et al. [24]	1	1	1	1	0	1	1	1	7
Lu et al. [33]	1	1	1	1	1	0	1	1	7
Shui et al. [34]	1	1	1	1	2	0	1	1	8
Kang et al. [25]	1	1	1	1	1	1	1	1	8
Zhou et al. [38]	1	1	1	1	2	0	1	1	8

a: Representativeness of the exposed cohort; b: Selection of the nonexposed cohort; c: Ascertainment of exposure; d: Illustration that there was no result of interest at start of research; e: Comparability of cohort based on the design or analysis; f: Evaluation of result; g: Was follow up long enough for outcome to happen; h: Adequate of follow up of cohort.

### Meta-analysis outcomes

#### One-year survival rate

**Fig 3:**
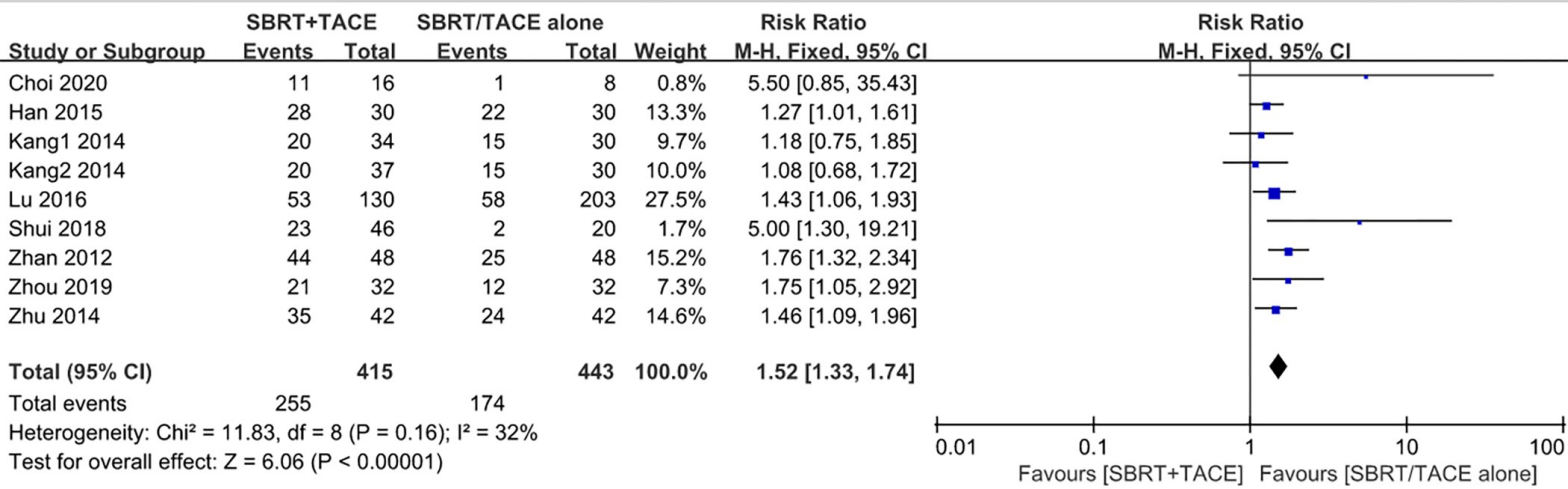
Forest plot of 1-year OSR in the SBRT plus TACE group versus monotherapy group for HCC with PVTT.

The 1-year survival rates were reported in 8 studies with 9 cohorts, including 415 patients in the SBRT+TACE group and 443 patients in the monotherapy group. Due to no statistically obvious heterogeneity existing among the studies (*I*^*2*^ = 32%; *P* = 0.16), a fixed-effects model was selected to analyse the 1-year survival rates. The pooled result revealed that SBRT plus TACE significantly improved 1-year survival rate compared with monotherapy(RR, 1.52 [95% CI, 1.33–1.74]) (Fig 3).

#### Two-year survival rate

**Fig 4:**
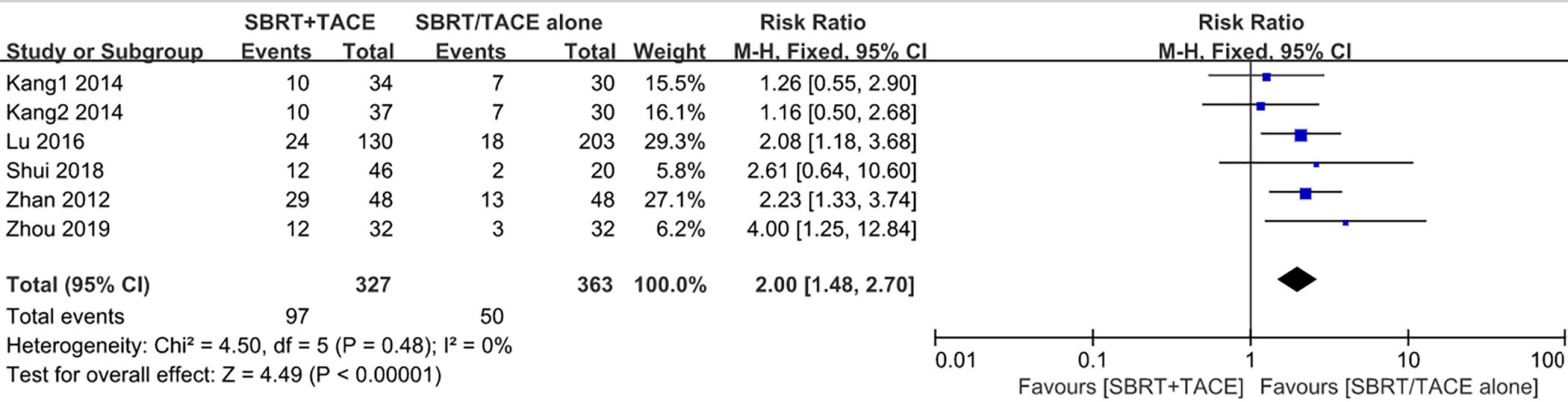
Forest plot of 2-year OSR in the SBRT plus TACE group versus monotherapy group for HCC with PVTT.

The 2-year survival rates were reported in 5 studies with 6 cohorts, including 327 patients in the SBRT+TACE group and 363 patients in the monotherapy group. A fixed-effects model was applied to analyse the results owing to no statistically significant heterogeneity across the studies (*I^2^* = 0%, *P* = 0.48). The pooled result indicated that the 2-year survival rate in SBRT plus TACE was significantly higher than that in monotherapy (RR, 2.00 [95% CI: 1.48–2.70]) (Fig 4).

#### Response rates

**Fig 5:**
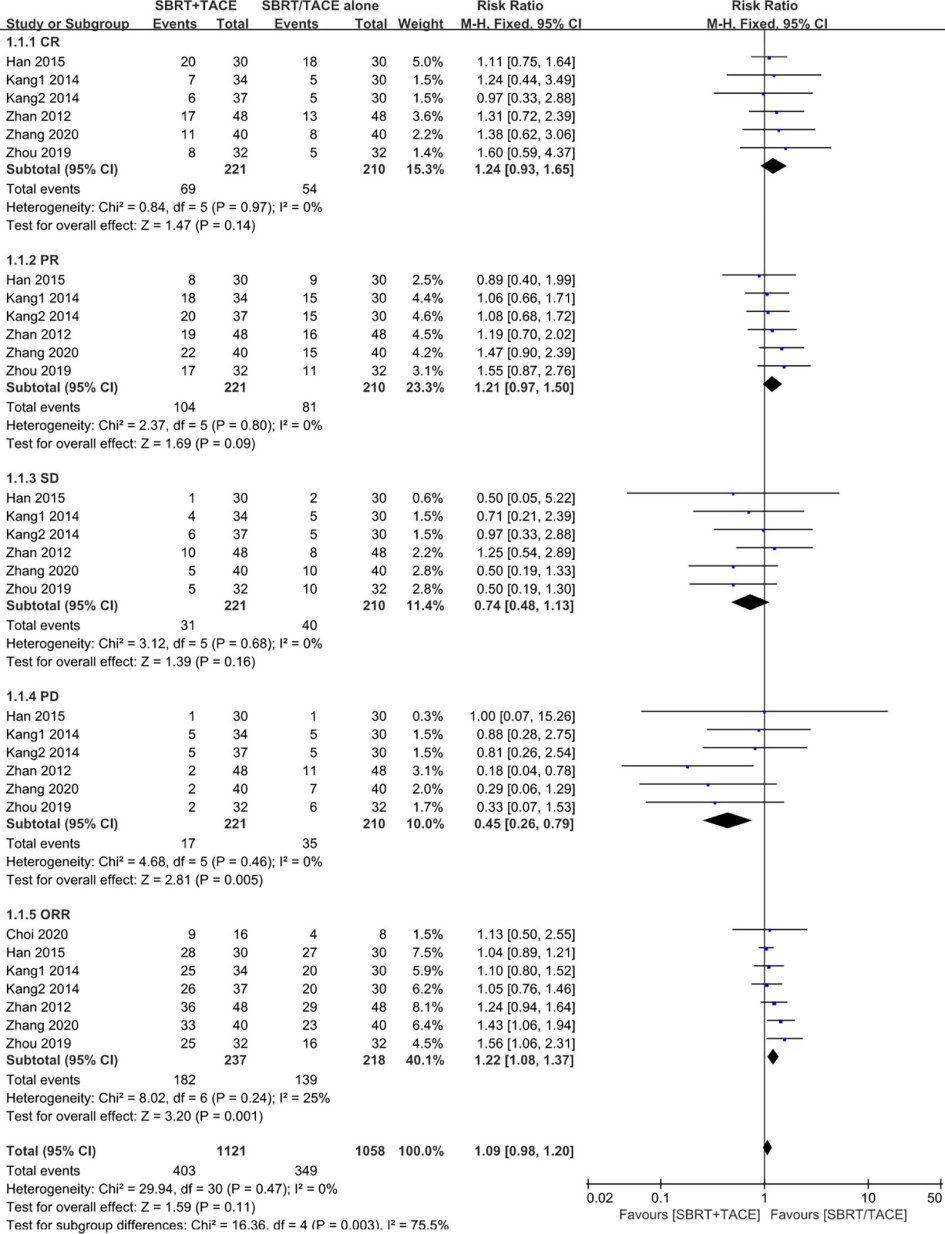
Forest plot of response rates in the SBRT plus TACE group versus monotherapy group for HCC with PVTT.

CR, PR, SD, and PD were reported in 5 studies with 6 cohorts but not in Choi et al.’s study [24], which presented only ORR. Heterogeneity in these studies was not significant, so a fixed-effects model was employed to pool the response rates to treatment. The results showed that SBRT plus TACE significantly improved the ORR of the target lesion in comparison with monotherapy (RR = 1.22 [95% CI: 1.08–1.37]). Moreover, combination therapy appeared to be strongly correlated with a lower rate of PD in comparison with monotherapy (RR = 0.45 [95% CI: 0.26–0.79]). However, no visible differences were found in CR, PR, and SD between the two groups (Fig 5).

### Subgroup analysis

#### Study type

By performing subgroup analysis regarding study type, we noticed that the combination of SBRT with TACE had higher 1- and 2-year survival rates than monotherapy in both RCTs and non-RCTs. Nevertheless, the difference of the ORR between the two groups was not significant in either RCTs or non-RCTs. The reason for this may be that a random-effects model was selected to merge the response rates to treatment owing to significant heterogeneity among the RCTs (*I*^2^ = 65%, *P* = 0.06) (Table 3).

**Table 3:** The subgroup meta-analysis of 1-year OSR, 2-year OSR, and ORR in regard to study type, control group, treatment order, SBRT-TACE interval between SBRT plus TACE group and monotherapy group.

Endpoint / Subgroup	Cohorts	No. of Studies	RR (95% CI)	*P* Value (Significance)	Heterogeneity	
					*I* ^*2*^	*P* Value
**1-year survival rate**		**9**	**1.52(95%CI,1.33–1.74)**	**<0.00001**	**32%**	**0.16**
**Study type**	**RCTs**	**3**	**1.51(95%CI,1.28–1.77)**	**<0.00001**	**37%**	**0.21**
	**Non-RCTs**	**6**	**1.53(95%CI,1.25–1.87)**	**<0.0001**	**42%**	**0.12**
**Control group**	**SBRT alone**	**6**	**1.45(95%CI,1.08–1.95)**	**= 0.01**	**58%**	**0.04**
	**TACE alone**	**3**	**1.48(95%CI,1.21–1.82)**	**= 0.0001**	**0**	**0.79**
**Treatment order**	**SBRT followed by TACE**	**5**	**1.59(95%CI,1.10–2.30)**	**= 0.01**	**66%**	**0.02**
	**TACE followed by SBRT**	**4**	**1.42(95%CI,1.18–1.70)**	**= 0.0002**	**0**	**0.57**
**SBRT-TACE interval**	**<28d**	**2**	**1.56(95%CI,1.20–2.02)**	**= 0.001**	**0**	**0.53**
	**≥28 d**	**3**	**1.30(95%CI,0.94–1.80)**	**= 0.12**	**35%**	**0.21**
**2-year survival rate**		**6**	**2.00(95%CI: 1.48–2.70)**	**<0.00001**	**0**	**0.48**
**Study type**	**RCTs**	**1**	**2.23(95%CI,1.33–3.74)**	**= 0.002**	**/**	**/**
	**Non-RCTs**	**5**	**1.91(95%CI,1.32–2.76)**	**= 0.0006**	**4%**	**0.39**
**Control group**	**SBRT alone**	**4**	**1.76(95%CI,1.21–2.57)**	**= 0.003**	**0**	**0.44**
	**TACE alone**	**2**	**2.42(95%CI,1.45–4.03)**	**= 0.0007**	**0**	**0.32**
**Treatment order**	**SBRT followed by TACE**	**3**	**1.97(95%CI,1.29–3.00)**	**= 0.002**	**0**	**0.48**
	**TACE followed by SBRT**	**3**	**2.03(95%CI,1.32–3.12)**	**= 0.001**	**34%**	**0.22**
**SBRT-TACE interval**	**<28d**	**1**	**4.00(95%CI,1.25–12.84)**	**= 0.02**	**/**	**/**
	**≥28 d**	**2**	**1.21(95%CI,0.67–2.18)**	**= 0.53**	**0**	**0.89**
**ORR (CR + PR)**		**7**	**1.22(95%CI: 1.08–1.37)**	**= 0.001**	**25%**	**0.24**
**Study type**	**RCTs**	**3**	**1.20(95%CI,0.95–1.52)**	**= 0.13**	**65%**	**0.06**
	**Non-RCTs**	**4**	**1.20(95%CI,0.99–1.46)**	**= 0.06**	**0**	**0.45**
**Control group**	**SBRT alone**	**5**	**1.12(95%CI,0.97–1.28)**	**= 0.12**	**0**	**0.82**
	**TACE alone**	**2**	**1.49(95%CI,1.17–1.89)**	**= 0.001**	**0**	**0.73**
**Treatment order**	**SBRT followed by TACE**	**4**	**1.13(95%CI,0.97–1.31)**	**= 0.11**	**0**	**0.64**
	**TACE followed by SBRT**	**3**	**1.33(95%CI,1.10–1.61)**	**= 0.004**	**29%**	**0.24**
**SBRT-TACE interval**	**<28d**	**2**	**1.49(95%CI,1.17–1.89)**	**= 0.001**	**0**	**0.73**
	**≥28 d**	**3**	**1.08(95%CI,0.87–1.36)**	**= 0.48**	**0**	**0.98**

#### Control group

A subgroup analysis with regard to monotherapy regimens in the control group showed that compared with either SBRT alone or TACE alone, SBRT plus TACE was able to significantly improve 1- and 2-year survival rates. In addition, SBRT plus TACE seemed to improve the ORR compared with TACE alone (RR = 1.49 [95% CI: 1.17–1.89], *P* = 0.001) but not compared with SBRT alone (RR = 1.12 [95% CI: 0.97–1.28], *P* = 0.12) (Table 3).

#### Treatment order

By conducting subgroup analysis regarding the treatment order, we found that patients receiving TACE followed by SBRT yielded a better ORR than those receiving monotherapy (RR = 1.33 [95% CI: 1.10–1.61], *P* = 0.004). Whereas patients receiving SBRT followed by TACE had no statistically significant difference in ORR compared with those receiving monotherapy (RR = 1.13 [95% CI: 0.97–1.31], *P* = 0.11). Regardless of the treatment order, the 1- and 2-year survival rates in the combination treatment group were significantly better than those in the monotherapy group (Table 3).

#### SBRT-TACE interval

For patients with SBRT-TACE interval less than 28 days, SBRT plus TACE seemed to be more effective than monotherapy for the 1- and 2-year survival rates and ORR (*P* < 0.05). Whereas, for patients with an SBRT-TACE interval equal to or longer than 28 days, no obvious distinctions were observed in 1- and 2-year survival rates and ORR between the two groups (*P* > 0.05). In other words, there was a more significant trend for patients with SBRT-TACE interval less than 28 days to have better long-term survival and objective response rates than for those with SBRT-TACE interval equal to or longer than 28 days (Table 3).

#### Adverse events

**Fig 6:**
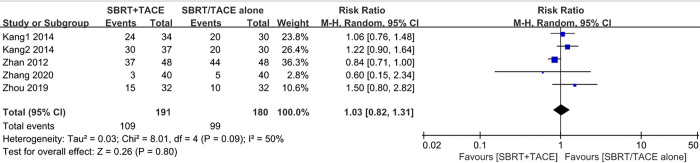
Forest plot of adverse events in the SBRT plus TACE group versus monotherapy group for hepatocellular carcinoma with PVTT.

Out of all eligible studies, 6 reported the occurrence rates of treatment-related AEs within 3 months after SBRT, mainly including bone marrow suppression, fever, hepatic toxicity, hepatalgia, anorexia, nausea and vomiting, and duodenum ulcer, most of which were mild to moderate (grade 1–2), with a very few grade ≥3. Almost all adverse events were alleviated or improved after active symptomatic treatment. No radiation-induced liver disease (RILD) was encountered in any HCC patients with PVTT within 3 months following SBRT. Furthermore, there were no AE-induced deaths in either group of patients, who were all restored to normal after treatment. No differences in the incidences of total AEs between the two groups were detected (RR = 1.03 [95% CI: 0.82–1.31], *p* = 0.80) (Fig 6). For each adverse event, the results showed no significant difference in the incidences of bone marrow suppression, fever, hepatic toxicity, hepatalgia, gastrointestinal reactions, or duodenum ulcers between the two groups of patients (Table 4).

**Table 4:** The meta-analysis of adverse events between SBRT plus TACE group and monotherapy group.

AEs	SBRT+TACE (event/total)	Monotherapy (event/total)	RR (95% CI)	p value	Heterogeneity	
					*I* ^*2*^	*p* value
**The total AEs**	**109/191**	**99/180**	**1.03(95%CI,0.82–1.31)**	**= 0.80**	**50%**	**0.09**
**Bone marrow Suppression**	**34/113**	**24/102**	**1.23(95%CI,0.79–1.90)**	**= 0.35**	**14%**	**0.31**
**Fever**	**30/113**	**29/102**	**0.90(95%CI,0.59–1.36)**	**= 0.61**	**30%**	**0.24**
**Hepatic toxicity**	**34/161**	**28/142**	**1.01(95%CI,0.66–1.54)**	**= 0.97**	**0**	**0.51**
**Hepatalgia**	**12/114**	**16/114**	**0.76(95%CI,0.39–1.47)**	**= 0.41**	**0**	**0.51**
**Gastrointestinal reaction**	**50/185**	**47/174**	**0.98(95%CI,0.70–1.35)**	**= 0.88**	**0**	**0.77**
**duodenum ulcer**	**3/72**	**0/72**	**4.00(95%CI,0.46–34.96)**	**= 0.21**	**0**	**0.82**

### Publication bias

Following the guidance of the Cochrane Handbook, an assessment of publication bias was unavailable as the number of studies included in each meta-analysis was less than 10.

## Discussion

PVTT in patients with HCC is one of the independent risk elements for poor overall survival [40,41]. Although a variety of treatment modalities, such as molecular targeted agents, surgery, TACE, and radiotherapy, have been confirmed to be effective for patients with PVTT, there is currently no consensus or recommendation on the optimal therapeutic modality for HCC with PVTT. So, we performed this meta-analysis for the evaluation of the efficacy and security of SBRT plus TACE for inoperable HCC with PVTT in comparison to monotherapy. We concluded that HCC patients with PVTT in the combined group had higher 1- and 2-year OSRs, ORR, and lower PD rates than those in the monotherapy group. No significant difference was found in terms of CR, PR, SD, or adverse events between the two groups of patients. With respect to study type, control group, and treatment order, subgroup analysis showed that compared with monotherapy, SBRT plus TACE greatly enhanced the 1- and 2-year survival rates but not the ORR. For the SBRT-TACE interval, subgroup analysis showed that patients with an SBRT-TACE interval <28 days had a higher 1- and 2-year OSRs and ORR than those with an SBRT-TACE interval ≥28 days.

There is, at present, considerable interest in the combination of RT with other treatment modalities, such as TACE, targeted therapy, or microwave ablation, which has been gradually recommended as an emerging treatment for HCC with PVTT and has been demonstrated to be superior to any single therapeutic regimen. Kim et al. [42] reported that the combination of RT and TACE in inoperable HCC patients with PVTT was preferred over TACE alone in terms of OS and time to progression (TTP) (OS, 11.4m vs. 7.4m; TTP, 8.7m vs. 3.6m). A meta-analysis conducted by Zhao et al. [43] evaluated the safety and effectiveness of SBRT plus TACE in comparison with SBRT alone as the first-line treatment for inoperable HCC. Their results presented that the combination therapy group had a higher disease control rate (DCR) and a longer OS than the monotherapy group in all patients but not in the subgroup of those with PVTT. The main reason for this may be that only three studies were included in their subgroup analysis for PVTT patients. Our meta-analysis involving nine studies concluded that SBRT combined with TACE had significantly higher 1- and 2-year OS and ORR than monotherapy. In general, patients who responded well to treatment had better survival benefits than those who responded poorly. Shui et al. [34] reported that the mOS of 12 months in patients receiving SBRT combined with TACE was significantly longer than the 3 months among those undergoing SBRT alone. Furthermore, the mOS was 13 months in HCC patients with a good response to PVTT and only 4.0 months in those without a response. Their result was in line with that of Yu et al. [44], who performed a single-arm clinical research on radiotherapy combined with TACE for HCC with PVTT. They concluded that the mOS in patients who responded well to treatment was 17.6 months, significantly higher than the 4.3 months in those who did not respond.

Subgroup analysis of the control group showed that SBRT plus TACE resulted in a higher ORR than TACE alone; however, there was no significant trend for patients treated by SBRT plus TACE to have a better ORR compared with patients treated by SBRT alone. This may be closely related to the direct killing or inhibitory effect of SBRT on tumor cells in portal vein in addition to intrahepatic tumour lesions. However, TACE has no killing effect on PVTT except for intrahepatic tumour cells. Few studies have focused on the treatment order of SBRT and TACE, the purpose of this subgroup analysis was to determine whether the prognosis of patients with PVTT could be influenced by the treatment order of SBRT and TACE. The results revealed that whether SBRT followed by TACE or TACE followed by SBRT, SBRT plus TACE was significantly better than monotherapy for 1- and 2- year survival rates of patients; however, compared with patients receiving TACE followed by SBRT, there was a nonsignificant trend for patients receiving SBRT followed by TACE to have a higher ORR than monotherapy. However, Kang et al. reported that there was no significant difference in ORR, 1- and 2- year survival rates between groups A (SBRT followed by TACE) and B (TACE followed by SBRT) [25]. Due to the small number of studies included, the reliability of the outcomes should be considered with caution. Therefore, it remains unclear whether patient outcomes are affected by the treatment order of SBRT and TACE.

In addition, we also found that the 1- and 2-year survival rates and ORR of patients with SBRT plus TACE were superior to those with monotherapy in the SBRT-TACE interval <28 days group; However, in the interval ≥28 days group, the combination therapy showed no greater survival benefit than monotherapy. Similar findings were also reported in the meta-analysis of Huo et al [45], who evaluated the efficacy and safety of TACE combined with radiotherapy compared with TACE alone in the treatment of unresectable HCC. Their results showed that in patients who had RT less than 28 days after TACE, RT plus TACE yielded less no response (NR) and better 3-year survival rates than TACE alone. However, this comparison was nonsignificant in patients who had RT 28 days or more after TACE. This may be because if the interval is too long, tumour cells proliferate rapidly, and the synergistic effect of SBRT and TACE cannot be fully exploited. Therefore, we suggest that the interval time between SBRT and TACE should be less than 28 days to achieve a better prognosis on the premise that it is safe and well-tolerated by the patients.

The location of the PVTT is also a well-known prognostic factor of patients with PVTT, with worse outcomes when it is in the main trunk [24,33,43]. Choi et al. [24] who evaluated the effectiveness of SBRT for HCC with PVTT concluded that patients with main trunk PVTT had a worse 1-year survival rate (54.7% VS. 75%) and a lower ORR (30% VS. 71.4%) than those with branch PVTT. What’s more, the rate of hepatotoxicity was higher in HCC patients with PVTT located in the main trunk than in those with PVTT located in the branches (40% vs. 14.3%). Hence, the best therapeutic regimen and radiotherapy dose shall be determined according to the location of the PVTT so as to achieve a better tumour remission rate while reducing hepatotoxicity. However, restricted by the insufficient data reported by the included studies, a separate subgroup analysis regarding PVTT classification could not be performed.

In regard to adverse events (AEs), we found that SBRT plus TACE had similar incidence of total AEs with monotherapy. For each treatment-related side effect, such as bone marrow suppression, fever, hepatic toxicity, hepatalgia, gastrointestinal reactions, and duodenum ulcers, there was also no significant difference between the two groups. However, some researchers pointed out that the combination of SBRT and TACE might exacerbate AEs mentioned above [25,43]. In general, treatment-related adverse events (TRAEs), especially 3–5 and AEs, may shorten patient survival in addition to reducing quality of life. Choi et al.’s study indicated that none of the patients with grade 3 or higher hepatotoxicity following SBRT survived for more than a year, while the 1-year survival rate could be as high as 81.1% in those without grade ≥3 hepatotoxicity [24]. But in fact, SBRT plus TACE did not result in a significant increase in the incidences of serious AEs, most of which could be alleviated or eliminated by early aggressive therapy. A meta-analysis performed by Zhao et al. [43] showed no significant difference in total AEs and grade ≥3 AEs between the two groups, aside from the slightly higher incidences of myelosuppression and fever in unresectable HCC patients undergoing SBRT plus TACE than in those undergoing SBRT alone. Limited by the insufficient data of the included literature, we were unable to carry out a separate analysis regarding grade ≥ 3 AEs. Overall, the combination therapy of SBRT and TACE is a secure and effective treatment for unresectable liver cancer with PVTT. Nevertheless, it is vital to select HCC patients with caution and closely monitor for potential AEs.

In our opinion, the clinical efficacy of SBRT combined with TACE is superior to that of a single treatment for the following reasons: 1) SBRT has the characteristic of precisely delivering a high intensity radiation dose to the target lesions and efficiently shrinking the tumour volume within a short time, which contributes to the recanalization of the portal vein, restoration of portal blood flow, regression of the arterioportal shunt, alleviation of hepatic ischaemia, and improvement of liver function, thereby providing better conditions for the subsequent TACE [40,46]; 2) TACE prior to SBRT can reduce the tumour volume and the corresponding radiation field, thereby increasing the dose in the tumour target area and reducing the radiation damage to adjacent normal tissues and organs; 3) SBRT after TACE may form a second blow to the target lesions, and then kill residual tumour cells after TACE, enhancing the curative effect, shortening the therapeutic process, and preventing the relapse and recoil [25]; 4) Chemotherapy drugs used in TACE can increase the sensitivity of tumour cells to radiation and further strengthen the lethal effect of radiotherapy on the target tissue. In general, SBRT and TACE play a coordinating role in killing tumour cells through different mechanisms of action [47,48].

### Study limitations

Our outcomes should be explained prudently in view of the limitations of the study. First, only 9 studies with 938 patients were included in our meta-analysis, and a smaller number of studies might affect the accuracy of the results. Second, among the nine studies, only four were RCTs, and three of them not reported allocation concealment. Double-blind was not described in any study in detail. All of the studies presented unclear risks in terms of blinding during the outcome assessment. In this case, the results were prone to be affected by selection bias, performance bias, and detection bias. Third, all of the studies were from Asia, with one from South Korea and the remaining eight from China. The aetiology of patients in Asia is different from that in the West to some extent, which is likely to result in regional bias. Finally, the baseline characteristics (e.g., tumour stage, Child-Pugh class, location of the PVTT, radiation dose, chemotherapy agents and the doses used in the TACE) of patients with HCC were not identical across the enrolled studies, which might affect the heterogeneity and the final results.

## Conclusion

Our meta-analysis offered compelling evidence that in unresectable hepatocellular carcinoma patients with PVTT, especially in those with SBRT-TACE interval <28 days, SBRT plus TACE was more effective than monotherapy in both long-term survival and short-term response rates. Moreover, the combination therapy was well-tolerated without a significant increase in the incidences of complications compared with monotherapy. Based on the above results, we suggest that SBRT plus TACE is a secure, efficient, and very hopeful treatment modality for inoperable HCC patients with PVTT. In the future, large multicenter RCTs are warranted to definitively confirm the security and effectiveness of SBRT combined with TACE in treating HCC patients with PVTT.

## Supporting information

S1 Checklist PRISMA checklist. (DOC)
